# Nanoscopic X-ray tomography for correlative microscopy of a small meiofaunal sea-cucumber

**DOI:** 10.1038/s41598-020-60977-5

**Published:** 2020-03-03

**Authors:** Simone Ferstl, Thomas Schwaha, Bernhard Ruthensteiner, Lorenz Hehn, Sebastian Allner, Mark Müller, Martin Dierolf, Klaus Achterhold, Franz Pfeiffer

**Affiliations:** 1 Chair of Biomedical Physics, Department of Physics and Munich School of BioEngineering, Technical University of Munich, 85748 Garching, Germany; 2 Department of Integrative Zoology, University of Vienna, 1090 Vienna, Austria; 3 Zoologische Staatssammlung München - SNSB, 81247 Munich, Germany; 4 Department of Diagnostic and Interventional Radiology, School of Medicine and Klinikum rechts der Isar, Technical University of Munich, 81675 Munich, Germany

**Keywords:** X-rays, Animal physiology, X-rays, Microscopy, Imaging techniques

## Abstract

In the field of correlative microscopy, light and electron microscopy form a powerful combination for morphological analyses in zoology. Due to sample thickness limitations, these imaging techniques often require sectioning to investigate small animals and thereby suffer from various artefacts. A recently introduced nanoscopic X-ray computed tomography (NanoCT) setup has been used to image several biological objects, none that were, however, embedded into resin, which is prerequisite for a multitude of correlative applications. In this study, we assess the value of this NanoCT for correlative microscopy. For this purpose, we imaged a resin-embedded, meiofaunal sea cucumber with an approximate length of 1 mm, where microCT would yield only little information about the internal anatomy. The resulting NanoCT data exhibits isotropic 3D resolution, offers deeper insights into the 3D microstructure, and thereby allows for a complete morphological characterization. For comparative purposes, the specimen was sectioned subsequently to evaluate the NanoCT data versus serial sectioning light microscopy (ss-LM). To correct for mechanical instabilities and drift artefacts, we applied an alternative alignment procedure for CT reconstruction. We thereby achieve a level of detail on the subcellular scale comparable to ss-LM images in the sectioning plane.

## Introduction

The relevant structures in biological specimens span over a wide range of length scales^1–3^. To obtain a holistic understanding of such structures, correlative microscopy combines multiple imaging techniques to investigate a single sample. It thereby exploits the specific strengths of the different imaging modalities, particularly with respect to sample sizes and resolution, to answer modern life science questions^1,2,4^.

Among microscopic techniques correlative light and electron microscopy (CLEM) has been found to be particularly powerful. Both imaging fields are highly complementary^5^. CLEM is a collective term for all different combinations of light and electron microscopy, ranging from fluorescence super-resolution microscopy to conventional bright field light microscopy and from focused-ion-beam scanning electron microscopy (FIB-SEM) over serial-block-face SEM (SBF-SEM) to transmission electron microscopy (TEM)^1,4–6^. Since light and electron microscopy techniques conventionally feature limited penetration depths through matter, they typically rely on sectioning or complete destruction of the sample to create 3D information of entire small biological specimens^1,7,8^. This often prevents isotropic resolution, and the resulting data are affected by deficiencies such as sectioning and alignment artefacts^1,4,5^.

X-ray computed tomography (CT) is a widely used tool for non-destructive 3D imaging with isotropic resolution^3,9,10^. Recently, high-resolution X-ray CT (terminology used in this study: microCT resolutions around 1 µm, NanoCT resolutions around 500 nm and smaller) has been integrated in a growing number of correlative studies^1,7,11–13^.

Combining different imaging methods, correlative microscopy is often associated with an array of complex sample preparation protocols, involving specific fixation and staining techniques^1,4,14^. Each additional processing step increases the workload as well as the risk of corrupting the sample microstructure and thereby the resulting data^2^. It is therefore highly relevant to minimize the complexity of sample processing.

Handschuh *et al*.^4^ successfully demonstrated threefold correlation of microCT, LM and TEM on a biological specimen which solely underwent conventional TEM sample preparation. Their work highlights the merits of integrating microCT into CLEM approaches^4^. However, the resolution limit of conventional laboratory-based microCT lies well above 500 nm^8^ and hampers visualizing the anatomy of tiny biological samples, which account for a majority of earth’s animal diversity^8^.

Based upon this study, we want to investigate the potential of an in-house-built laboratory-based NanoCT setup for correlative microscopy of a specimen prepared for TEM^15^. Combining small X-ray focal spots with cone beam geometry, this NanoCT setup reaches resolutions down to 100 nm and is highly versatile respective to sample sizes^10,16,17^.

Meiobenthic organisms are relatively small organisms reaching body sizes of approximately 1 mm and are often found in sandy sediments^18^. Due to their size they are well suited for entire specimen sectioning but are difficult to image with sufficient resolution using conventional microCT. Hence, for this study we chose a meiofaunal sea cucumber, *Leptosynapta* cf. *minuta* (*L*. cf. *minuta*).

Previous studies have shown that our NanoCT setup is capable of analysing the proper morphology of critical-point dried (CPD) and agarose-embedded biological samples^8,10,19^. It was further shown that osmium tetroxide staining combined with CPD creates satisfactory contrast in our NanoCT data^10^. In the present study we acquired NanoCT scans of *L*. cf. *minuta*, which was osmium tetroxide fixed for TEM and embedded into resin. So far, no data of resin-embedded samples has been acquired with this setup.

Imaging of resin-embedded samples poses no problem for conventional microCT^4^. For NanoCT imaging, however, it raises new challenges particularly for correction of mechanical instabilities and thermal drift effects during CT reconstruction. Here, we applied an iterative method for estimating geometry parameters based on a metric recently developed for blind deconvolution in CT reconstruction^20^. Based on the NanoCT data, we were able to segment the internal organ system of the specimen. To evaluate the benefits of NanoCT imaging for correlative microscopy, we compare our reconstructed NanoCT volume data with a volume reconstruction generated with serial sectioning light microscopy (ss-LM). In the scope of these analyses, we perform a qualitative assessment of the contrast-to-noise ratio (CNR) and the resolution of the data sets.

Beyond offering new insights into the micromorphology of *L*. cf. *minuta*, our work illustrates the benefits laboratory-based NanoCT offers as a complementary tool for correlative microscopy. In particular, the combination of NanoCT and TEM promises great potential for multiscale imaging as an alternative to CLEM approaches for addressing specific scientific questions.

## Results

**Figure 1:**
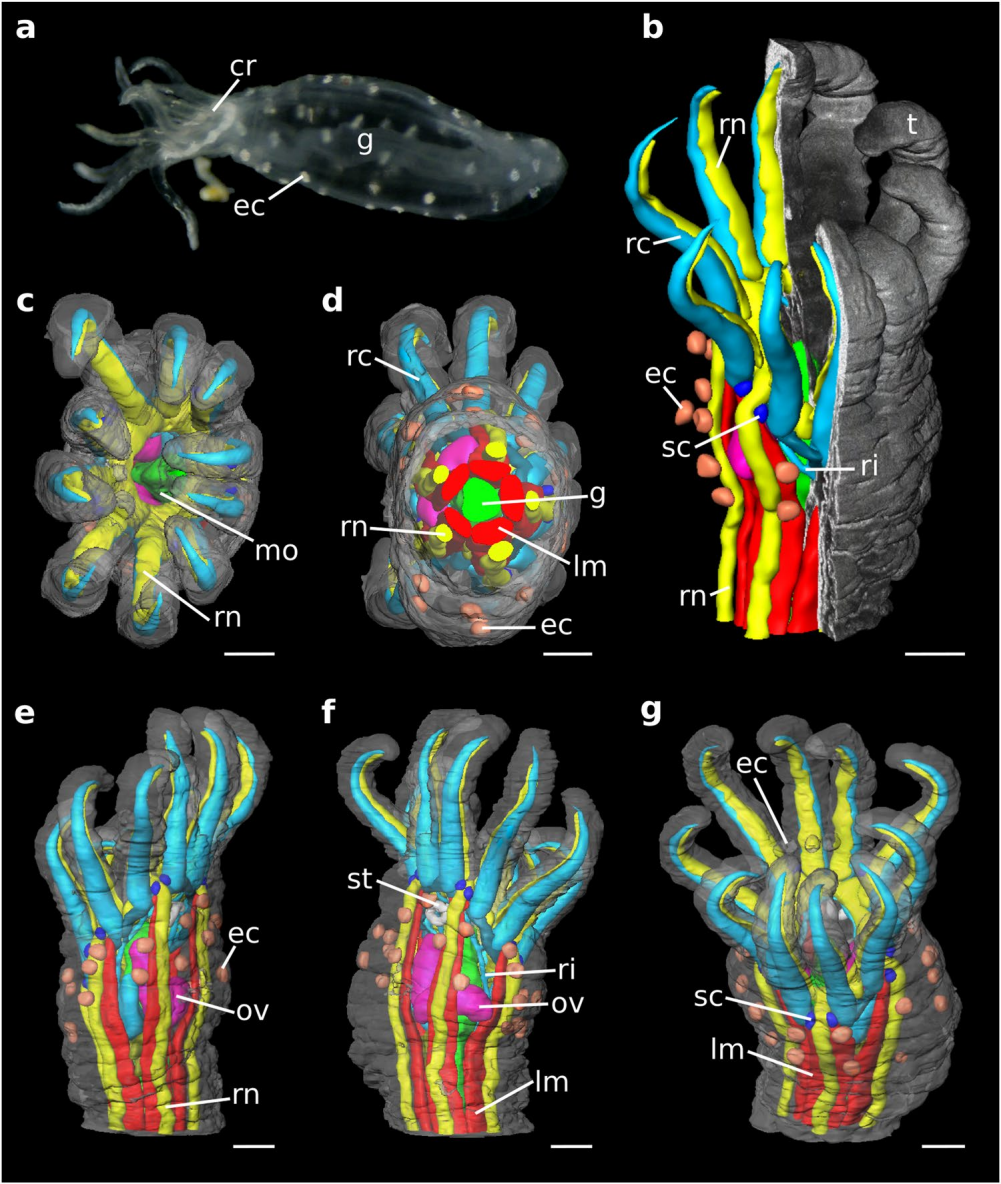
Morphology of *Leptosynapta* cf. *minuta*. Photograph (**a**), NanoCT volume renderings (effective voxel size ~540 nm) (**b–g**). (**a**) Overview over the alive specimen with an approximate body size of 1 mm. (**b**) Virtual section through the NanoCT 3D volume rendering exposing the segmented inner organs. (**c–g**) NanoCT 3D renderings depicting the body and the segmented organs in anterior (**c**), posterior (**d**), lateral (**e**), obliquely dorsal (**f**), ventral (**g**) views. Legend: cr: calcareous ring, ec: epidermal cups, g: gut, lm: longitudinal muscle, mo: mouth opening, rc: radial canal in tentacle, ri: ring canal, rn: radial nerve, sc: statocyst, st: stone canal, t: tentacle, ov: ovary. Scalebars: 100 µm.

With our in-house-built NanoCT setup, we analyzed a specimen of *Leptosynapta* cf. *minuta* which is an only little investigated small representative of a sea cucumber (Fig. 1a, Movie S1), adapted to a meiofaunal lifestyle. It is elongated and of ~1 mm length.

The sample was prepared for TEM imaging. It was post-fixated with osmium-tetroxide and embedded into resin. The NanoCT data was acquired of the entire specimen without sectioning. Thereby, the reconstructed CT volume provides isotropic resolution and preserves the 3D proportions of the sea cucumber (Fig. 1b–g).

For an overview of the animal, an adequate effective voxel size of 540 nm was chosen to minimize acquisition times. In this case, a centre shift correction was sufficient to achieve the best possible image quality. The osmium tetroxide post-fixation binds to lipids and proteins and thereby acts as an overview stain in X-ray imaging^4,10,21^. It generated an adequate overall contrast to access the 3D morphology of the sea cucumber. Thereby, the NanoCT volume provides a detailed representation of the 3D microstructure and allowed for modelling of the entire organ system of the specimen (Fig. 1b–g, Movie S2).

The trunk of the worm-shaped specimen appears mainly smooth, lacking any distinct surface structures except few papillary epidermal, sensory fields (Fig. 1). The anterior end of *L*. cf. *minuta* is characterized by ten tentacles (5 × 2) (Fig. 1b), which represent modified ambulacrary feet, surrounding the terminal mouth opening. The tentacles are slightly curved with their distal tips bent inwards. Based on the NanoCT data, we were able to determine the mean approximate dimensions of the tentacles, which result in a length of ~350 µm and a width of ~90 µm. On the side of the bivium (‘dorsal’, radia of C, D), three distinct epidermal cups are present (Fig. 1g).

The anterior mouth opening (Fig. 1c) continues into the digestive tract (Fig. 1d), which is a simple tube that traverses the entire trunk towards the posterior end, where it terminates via the anus. Apart from the digestive tract, the trunk mainly contains five prominent bundles of longitudinal muscles and five adjacent radial neurite bundles, together lying in the radia of the original pentamerous symmetry (Fig. 1d). Anteriorly, the longitudinal muscle bundles attach to the calcareous ring, which is bordered by the ring canal of the hydrocoel (RCH) (ambulacral system) (Fig. 1a,f). Due to its decalcified state and the stronger general soft-tissue contrast in the NanoCT data, the calcareous ring below the RCH is only slightly distinguishable in the NanoCT data and clearer on the ss-LM images (Fig. S1a,b). Five pairs of radial canals (Fig. 1b,d) emanate distally from the RCH into each pair of tentacles. On the proximal side, a short ampullary widening extends posteriorly on the outer side of the calcareous ring.

**Figure 2:**
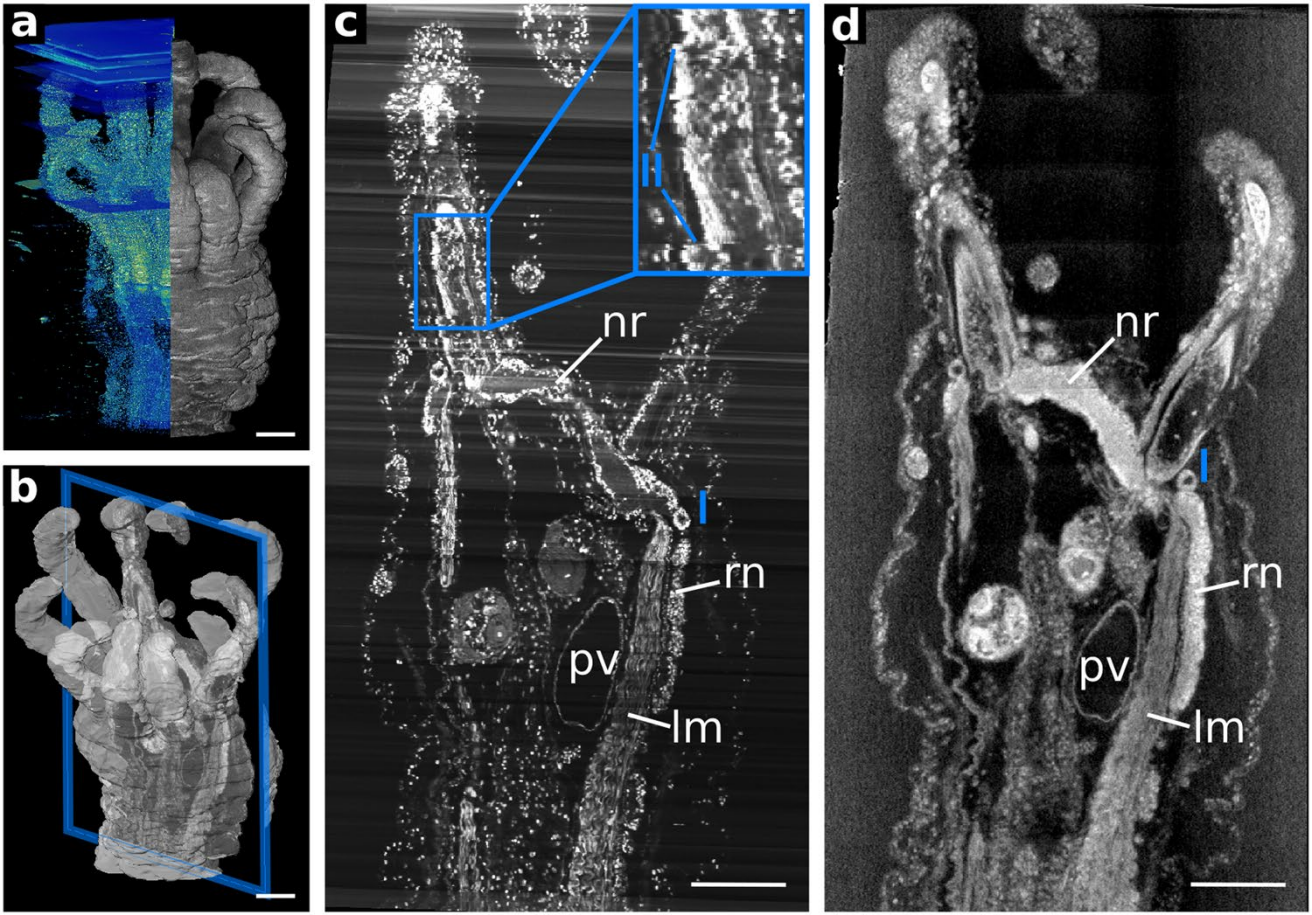
Co-registration of overview data from ss-LM (10x objective lens) and NanoCT (effective voxel size ~540 nm). Note that the grey values of the histological section images are inverted for better comparison. (**a**) 3D renderings of ss-LM data (left, blue/green) and NanoCT (right, grey) displayed adjacent to each other. (**b**) Plane of interest, which is depicted in (**c,d**), outlined in the volume rendering. (**c**) Virtual section of the ss-LM after alignment through the plane marked in (**b**). (**d**) Corresponding NanoCT slice through the same plane shown in (**b**). Legend: I: co-registration mismatch, II: alignment artefact, lm: longitudinal muscle, nr: nerve ring, pv: polian vesicle, rn: radial nerve. Scalebars: 100 µm.

**Figure 3:**
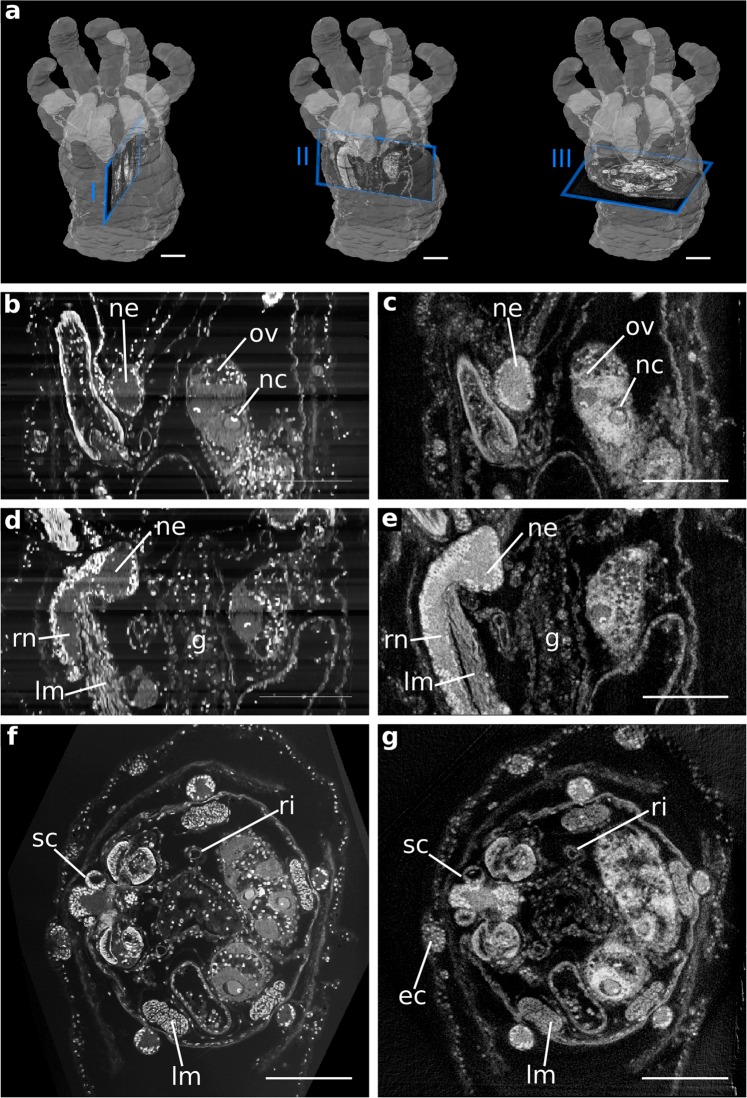
Comparison of high-resolution data from ss-LM (20x objective lens) and NanoCT (effective voxel size ~290 nm). Note that the grey values of the histological section images are inverted for better comparison. (**a**) Planes of interest, which are displayed in (**b–g**), outlined in the volume rendering. From left to right: I (shown in **b,c**), II (shown in **d,e**), III (shown in **f,g**). (**b**) ss-LM slice through plane I shown on the left in (**a**). (**c**) Corresponding NanoCT slice through plane I. (**d**) ss-LM slice through plane II displayed in (**a**). (**e**) Corresponding NanoCT slice through plane II. (**f**) ss-LM slice through plane III shown in (**a**). (**g**) Corresponding NanoCT slice through plane III. Legend: ec: epidermal cups, g: gut, lm: longitudinal muscle, nc: nucleolus, ne: nervous system, ov: ovary, ri: ring canal, rn: radial nerve, sc: statocyst. Scalebars: 100 µm.

The ring canal (and thus the calcareous ring) is accompanied by a prominent nerve ring (Fig. 2c,d), which anteriorly interconnects each pentameral radial nerve. Likewise, thick tentacle neurite bundles emerge from the nerve ring into each tentacle. These are located medially from the hydrostatic feet tube coelom. Paired statocysts (Fig. 1b,g) are located at the base of each pair of the oral tentacles. Within the statocysts, distinct statoliths are recognizable in the NanoCT and the ss-LM images, which are depicted in Fig. 3g.

Internally, approximately located in radius E, the polian vesicle (Fig. 2c,d) extends from the RCH largely into the body cavity. Likewise, a single stone canal (Fig. 1f) including a madreporite head can be found in interradius CD. The female gonad (Fig. 1e,f) containing large oocytes can be found in a similar position. Single oocytes including cellular details are discernible in the NanoCT and the ss-LM images (Fig. 3b,c). Posterior of the proximal end of the polian vesicle, a large vibratile urn (Fig. S1c,d) projects into the coelomic cavity.

To evaluate our methodology, we compare our NanoCT data to a ss-LM volume with a 10x objective lens, obtained from the same specimen. In Fig. 2 (and Movie S2) the co-registered ss-LM and NanoCT data, with the ss-LM grey levels inverted for better comparability, are shown.

The 3D co-registration of the two volumes in Fig. 2a appears consistent. Investigating a single plane, outlined in Fig. 2b, and the corresponding virtual slices (Fig. 2c,d**)**, however, reveals slight discrepancies. The most prominent mismatch is apparent at the right statocyst (Fig. 2c,d I). Nevertheless, Fig. 2c, and Fig. 2d depict sufficiently similar structures to assess the data quality of both volumes. In the ss-LM volume (Fig. 2a, left) and the ss-LM slice (Fig. 2c), planar or line shaped artefacts are visible originating from brightness deviations during LM acquisition. These artefacts indicate the sectioning planes in the series. In contrast to the NanoCT data, the ss-LM slice suffers from alignment artefacts (Fig. 2c II), which are common in physical sectioning-based techniques.

The ss-LM and the NanoCT slices exhibit different distributions of grey values (Fig. 2c,d). This originates from both the different physical imaging behaviour of ss-LM and NanoCT, and the specific stains, that were applied in this particular case. Despite the additional staining with methylene blue-azure II for the ss-LM data, the NanoCT data appear to display, here, even stronger overall soft-tissue contrast. Due to the high spatial coherence of the X-ray tube and the geometry of the NanoCT, propagation phase effects caused by Fresnel diffraction can be used to improve the overall contrast of the NanoCT data^22^. These phase effects are stronger at interfaces from air to sample material than from resin to sample. Therefore, by embedding the specimen into resin these contributions are reduced considerably. Furthermore, in the X-ray regime, the attenuation behaviour of stain and resin are more similar than for visible light. Nevertheless, the contributions of the phase effects are strong enough to compensate for this effect. This leads to an adequate overall soft-tissue contrast in the NanoCT data to perfectly reproduce the specific structures in the specimen.

To gain more detailed information of a volume of interest (VOI) (Fig. 3a), we acquired two more data sets with ss-LM and NanoCT with double magnification (ss-LM: 20x objective lens, NanoCT: effective voxel size ~290 nm). For such voxel sizes, a conventional centre shift correction does not suffice, and we applied an advanced alignment method.

In the high-resolution data (Fig. 3b-g, Movie S3), single muscle fibre bundles can be distinguished, and single cell nuclei are visible as bright dots. Beyond that, our findings reveal the precise shape of the nucleoli in the oocytes (Fig. 3b-g) and our NanoCT data resolve the statolith within the statocyst (Fig. 3g).

The ss-LM (Fig. 3b,d,f) and the NanoCT data (Fig. 3c,e,g) are well co-registered, with only minimal mismatches. Furthermore, the intensity artefacts parallel to the sectioning plane, which are rather pronounced in the overview ss-LM volume (Fig. 2c), appear only minimally in the partial volume used for co-registration with the high-resolution NanoCT data (Fig. 3b,d).

**Figure 4:**
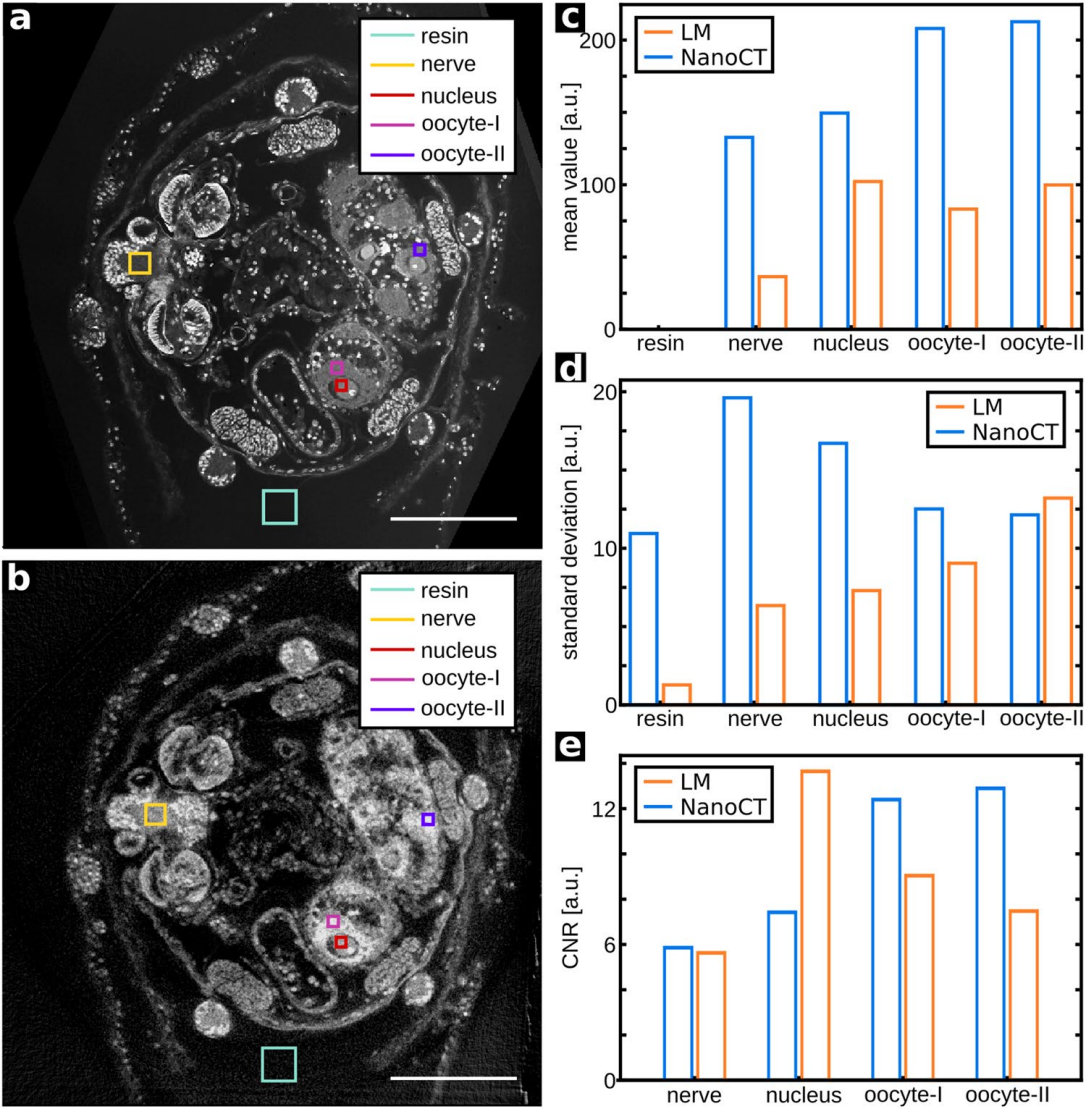
Comparison of the CNR of NanoCT and LM. (**a**), (**b**) LM section image and corresponding high-resolution NanoCT slice (effective voxel size ~290 nm) with different ROIs. (**c**,**d**) Bar chart of the mean value and the standard deviation of the LM and NanoCT data within the ROIs shown in (**a**,**b**). For reference the mean value of resin was set to 0. (**e**) CNR values with resin as reference of the LM and NanoCT data within the ROIs in (**a**,**b**). Scalebars: 100 µm.

As for the overview data, the different properties of the two imaging modalities and the specific staining techniques lead to a different distribution in grey values (Fig. 4). While the NanoCT data provides generally higher contrast values (Fig. 4c), it also tends towards higher noise levels (Fig. 4d). Consequently, the imaging modality providing the superior CNR strongly depends on the tissue type (Fig. 4e). For features such as the nuclei of the oocytes, where both data sets display similar contrast levels, the CNR value of the LM data is significantly higher. In contrast to that, the NanoCT delivers a better CNR in the cytoplasm of the oocytes. Comparing the results of two ROIs ‘oocyte-I’ and ‘oocyte-II’ of the same tissue type, the great influence of the choice of ROI becomes apparent. This should be considered in the evaluation of these results. Moreover, Fig. S2 illustrates the effect of retrieving the propagation-based phase information on the reconstructed slices. Due to the resin-embedding the majority of phase contributions are lost. Nevertheless, under the homogeneity assumption, the phase retrieval algorithm works as a boost in the contrast to noise ratio without losing resolution^23^.

**Figure 5:**
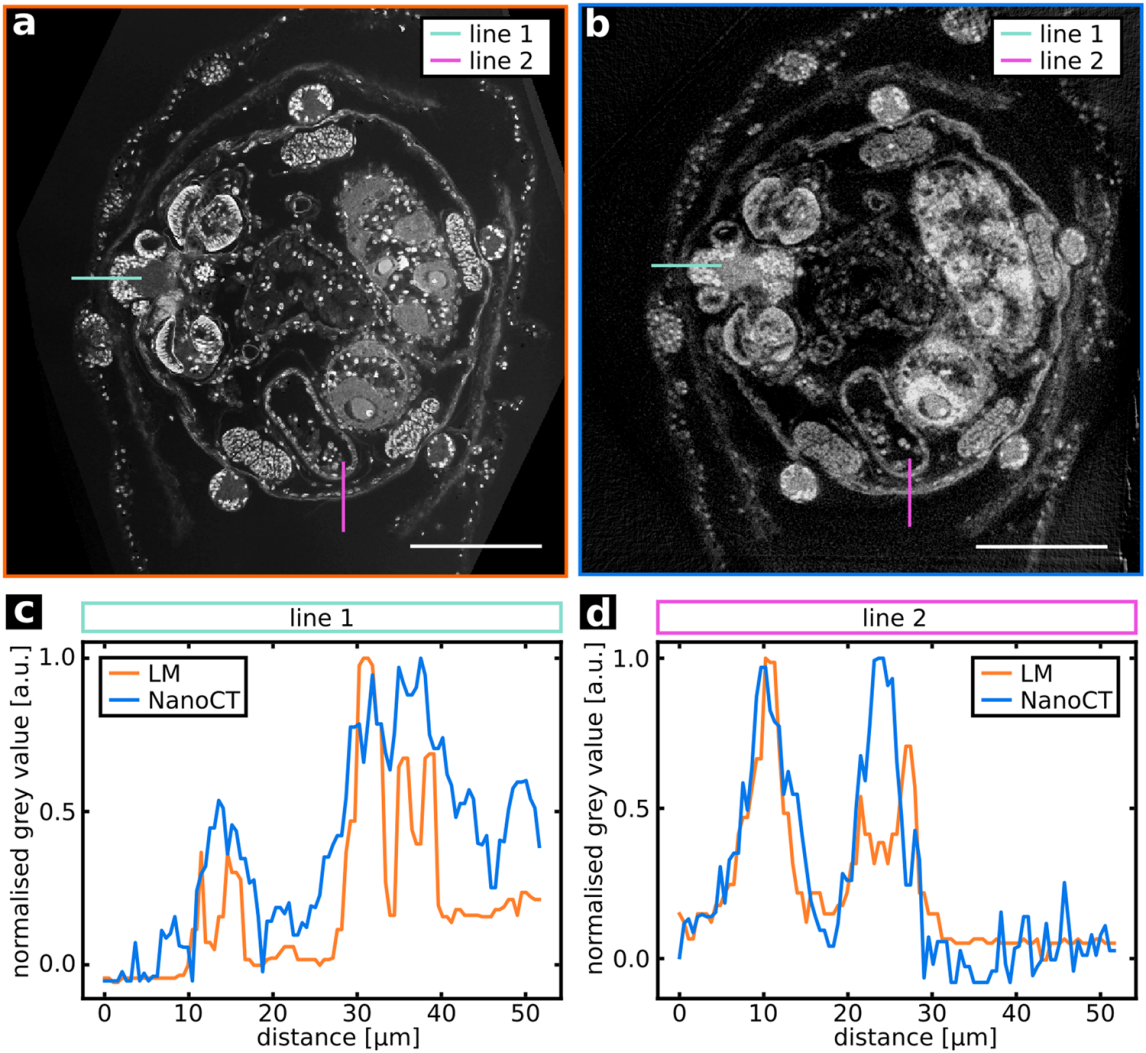
Comparison of the spatial grey value profile along two lines in the NanoCT and LM data illustrating the resolution of the two imaging modalities. (**a**,**b**) LM section image and corresponding high-resolution NanoCT slice (effective voxel size ~290 nm) with the chosen lines indicated. (**c**,**d**) Spatial distribution of the grey values along line 1, line 2 in the LM and NanoCT data. Scalebars: 100 µm.

In the sectioning plane (Fig. 3f,g), where the ss-LM image quality reaches its optimum, the NanoCT slices exhibit almost the same level of detail as the LM slices. This is more closely investigated in Fig. 5, which displays the grey value distribution along corresponding lines in the NanoCT slice and the ss-LM image from Fig. 3f,g. These distributions allow for a qualitative comparison of resolutions of the NanoCT and ss-LM in the sectioning plane. A quantitative assessment would require an ideal edge to derive the edge spread function and from that the image resolution^24^. The LM data in Fig. 5c,d exhibit steeper edges and more pronounced peaks, which originate from cell nuclei. This reflects observations likewise evident in Fig. 3f,g, where the cell nuclei appear more distinct in the LM image. Nevertheless, the NanoCT data coherently describe the features shown in the LM data, albeit with slightly less depth of detail. In contrast to that, the NanoCT data provides superior image quality in all spatial planes, which deviate from the sectioning plane, as it is free of alignment artefacts and provides isotropic resolution (Fig. 3b-e).

**Figure 6:**
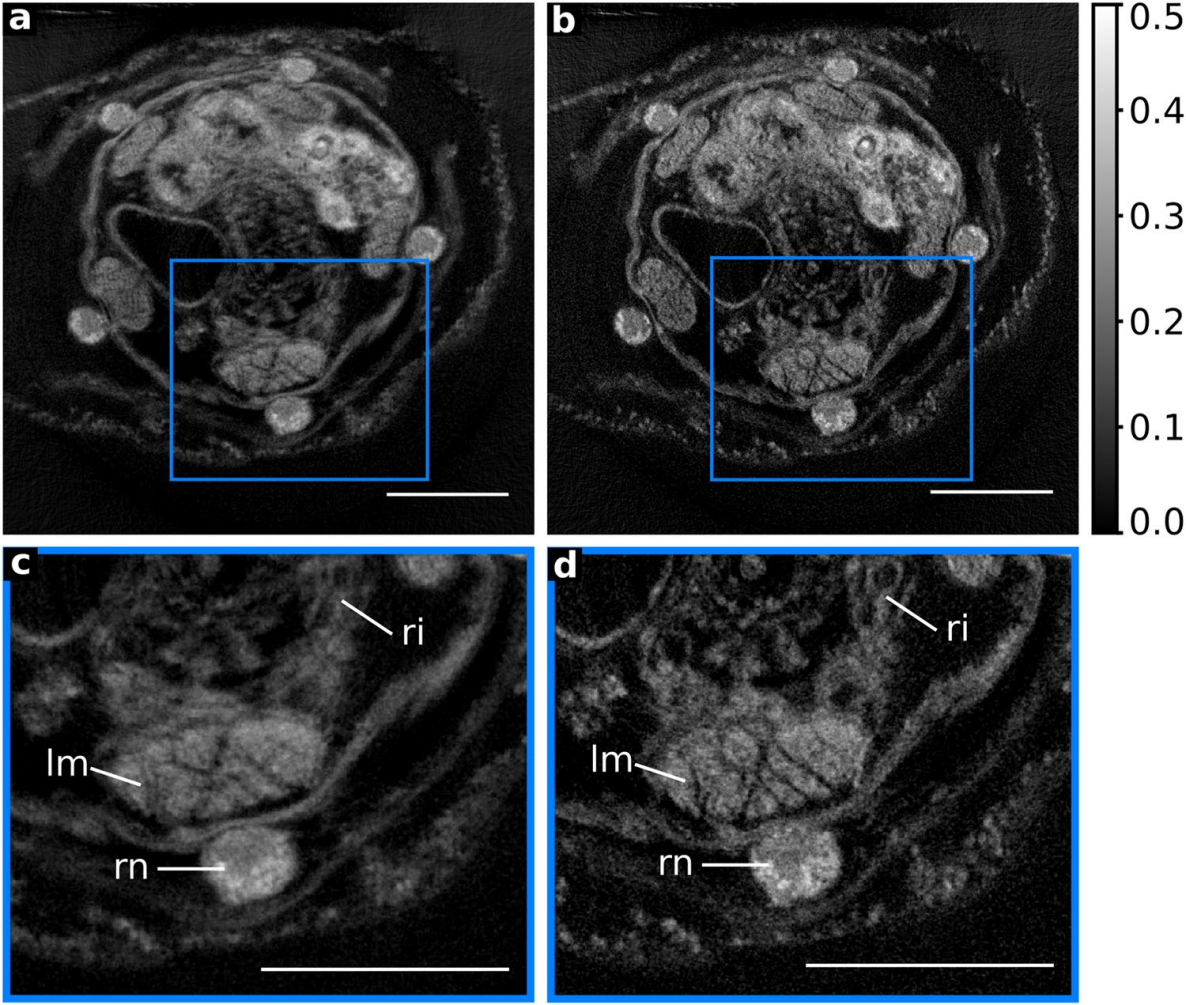
Comparison of filtered-back-projection (FBP) reconstructed high-resolution NanoCT data (effective voxel size ~290 nm) with either conventional centre shift correction or the novel alignment metric. (**a**) Centre shift corrected FBP reconstructed slice of the high-resolution NanoCT data. (**b**) FBP reconstructed slice using the advanced alignment metric of the high-resolution NanoCT data. (**c**) Detail image of the blue marked ROI in (**a**). (**d**) Detail image of the blue marked ROI in (**b**). Legend: lm: longitudinal muscle, ri: ring canal, rn: radial nerve. Scalebars: 100 µm.

Correctly accounting for the system geometry is crucial for the quality of the CT reconstruction. In the magnification regime of microCT, conventional centre shift correction, where the data is corrected for a global offset of the rotation axis from the optical axis, is often sufficient^25,26^. For NanoCT imaging, with much smaller effective voxel sizes (~290 nm), the previously mentioned mechanical instabilities have a much larger impact and diminish image quality significantly (Fig. 6a,c)^27^. Therefore, the shifts have to be corrected for every view individually requiring more advanced algorithms. Iterative alignment methods exist that use cross-correlation techniques to estimate the positional offset for every view^27–30^. However, cross-correlation methods work best, if the projections have high-contrast features and if the sample size does not exceed the field of view (FOV). Due to the resin-embedding, the contrast provided by phase effects is reduced and the noise levels are higher. Furthermore, these alignment methods are based on a parallel beam assumption. The conceptual design of the NanoCT, which is inherently associated with a wide cone-beam angle, usually doesn’t fulfil this assumption. Thus, for this NanoCT data set of a resin-embedded specimen, an alternative alignment algorithm was devised and compared to a centre shift correction. Our approach is based on a sparsity metric recently developed for blind deconvolution in CT reconstruction^20^. Thereby, the detector offset is optimized iteratively for every view until the reconstruction employs most sparsity according to the metric.

Figure 6 provides a comparative display of conventional centre shift correction (Fig. 6a,c) versus the novel alignment method (Fig. 6b,d). In contrast to the centre shift corrected slice (Fig. 6c), the NanoCT slice with the new alignment routine clearly visualizes the ring canal, the structures in the muscle fibre bundles and the nerve strands including the cell nuclei (Fig. 6d).

## Discussion

The non-destructive nature of X-ray CT enables further analysis with other imaging techniques. Hence, microCT has been integrated increasingly in correlative microscopy applications, to define VOIs and as a reference for sectioning-based microscopy^4,13,31^. But to genuinely gain information about the microstructure of tiny biological specimens, the resolution of conventional microCT does not suffice^8^.

Our table-top NanoCT setup reaches resolutions around 100 nm^10^. In contrast to other laboratory devices with comparable resolutions, it foregoes any light or X-ray optics^3^. As a result, the NanoCT has a broad energy bandwidth available for imaging and the magnification can be adjusted continuously^16^. Furthermore, it offers a larger FOV (540 nm effective pixel size: FOV ~ 796 µm x 105 µm, 290 nm effective pixel size: FOV ~ 428 µm x 56 µm) and stitching allows further extension of the FOV parallel to the rotation axis. This provides the NanoCT with a high versatility regarding sample sizes and makes it attractive for various microscopy applications. Here, these features enabled us to image a resin-embedded soft-tissue sample with dimensions of 1 mm × 0.5 mm, with the surrounding resin exceeding the sample dimensions even further. Unlike many other X-ray microscopes, the NanoCT allowed for measuring the sample in its entirety at different magnifications, without cutting the sample (Figs. 1–6**)**^7^.

The specimen was solely prepared with the standard TEM sample processing routine. This minimizes the workload and reduces the risk of processing artefacts. Furthermore, it is easily feasible to add a subsequent TEM analysis to our work-flow, enabling multi-scale imaging of one specimen^4^. Despite the advantages of imaging resin-embedded samples, it yields new obstacles to overcome for our NanoCT device. The relatively large size even of trimmed embedded samples complicates sample alignment for CT acquisition in the set-up. In contrast to LM, the resin’s X-ray absorption behaviour is not negligible. To reach satisfactory low noise levels, longer exposure times are required. Longer exposure times, however, are associated with more artefacts due to mechanical instabilities, and thermal drift effects, which can impair the results. Furthermore, it has to be noted that despite the risk of processing artefacts, additional stains and imaging modalities may also yield additional information^6^.

Osmium tetroxide provides an adequate general staining of the specimen’s micromorphology in the NanoCT data (Figs. 1–6). Retrieving the propagation phase information in the NanoCT data further enhances the soft-tissue contrast (Fig. S2), resulting in adequate CNR levels (Fig. 4). Depending on the type of tissue, the NanoCT enables even better CNR values than ss-LM in the sectioning plane (Fig. 4e). Apart from trimming the resin-block containing the specimen for a minimal sample size, the NanoCT does not require additional sample processing. Nevertheless, the NanoCT data allowed for accessing the micromorphology of the sea cucumber and enabled 3D modelling of the entire organ system (Fig. 1). Throughout this process, only a single type of organ was difficult to distinguish in the overview NanoCT data, the vibratile urn (Fig. S1d), and this structure was not included in the VOI of the high-resolution NanoCT data. Here, further information could be gained with ss-LM (Fig. S1c).

Our findings yielded the general morphology of *Leptosynapta* to be similar to other holothurians with a bottom-facing (or ‘ventral’) trivium comprising radia E, A, B and the upper bivium of radia C, D. Between the latter lies the stone canal and the opening of the gonad (cf.) (Fig. 1f). Apodan holothurians are a clade of holothurians lacking radial water canals (of the hydrocoel) and thus (namely) also the radial rube feet^32^. They form the sister-group to the remaining, feet-bearing, Actinopoda (e.g.^33^). Several morphological details of the genus *Leptosynapta* have been previously analysed. This includes statocyst structure^34^, anal pores^35^, ciliated cups^36^, ossicle formation^37^, tentacle ultrastructure^38^ and sperm structure^39^.

With few exceptions, such as the synaptids, the digestive tract in holothurians is generally long and coiled^40^. As previously reported^41,42^, the gut in *L*. cf. *minuta* is shaped as a simple straight tube (Fig. 1d). Furthermore, our data reveal a vibratile urn (Fig. S1c,d). In contrast to other species, where vibratile urns appear in higher numbers^32^, *L*. cf. *minuta* exhibits only one. Vibratile urns are trumpet- to cornet-shaped coelomic organs that act in circulation and coelomic clearance and were studied in detail in *L. inaherens*^43^. While these structures have already been found in some members of the genus *Leptosynapta*, they are unique for synaptid holothurians. In contrast to that, epidermal sensory structures are common for many synaptid holothurians and are considered a compensation for the lack of tube feet^36^. As displayed by Fig. 1, they are regularly distributed over the anterior body, but are also present as three cups on the inner side of the bivium. Beyond that, our results show statocysts surrounding the mouth opening (Fig. 1b,g). They have been previously described for the genus^44^ and other apodous holothurians^32^ and are regarded as adaptations to a burrowing lifestyle^45^. Detailed descriptions of their structure are provided by Ehlers^34^.

In a comparative analysis with ss-LM, which is widely used for correlative zoological studies^4^, we evaluated our NanoCT data. In contrast to ss-LM, the NanoCT is non-destructive, provides isotropic resolution and preserves the correct proportions of the resin-embedded sample, without the interference of distortion or alignment artefacts. It can, therefore, be used for length or volume calculations and as a reference for sectioning-based microscopy to retrieve the correct sample proportions. Here, these advantages were exploited to calculate the dimensions of the tentacles and the NanoCT data was used as a reference to obtain the correct ss-LM volume for Figs. 2 and 3. In the sectioning plane, the NanoCT slices show fine structures, such as the statolith in the statocyst (Fig. 3g), single cell nuclei and the specific shape of the nucleoli in the oocytes (Fig. 3c), comparable to the ss-LM images. Nevertheless, the resolution of ss-LM in the sectioning plane is higher in the presented data, as illustrated in Fig. 5. In the case of *L. cf. minuta*, though, the slightly enhanced level of detail in the ss-LM sections on the cytological level hardly yields any additional benefit for the understanding of the sea cucumber’s morphology. Alignment as well as intensity artefacts (Figs. 2c and 3b,d) substantially reduce the image quality of the ss-LM slices in any arbitrary virtual plane other than the sectioning plane. In these planes, as shown in Fig. 3c,e, the quality of the NanoCT data is significantly higher.

When weighing the benefits of the two methods, the amount of work involved, and the usability are not to be neglected. Investigating the acquisition times of the two imaging modalities, the NanoCT acquisition is with around 30 h for each of the presented volumes significantly longer than the time required for an ss-LM image stack, which lies in the range of 1 h–2.5 h. It has to be considered, however, that the actual CT acquisition is automated. The time to prepare an automated CT acquisition takes an operator around 3 h. Therefore, the image acquisition of both methods can be regarded as similarly labour intensive. Due to the novelty of the NanoCT system, no commercial image processing software is available to obtain adequate results and the applied algorithms are still rather complex compared to ss-LM. The true power of ss-LM though lies within the wealth of available staining methods. While a lot of research is done on the implementation of new X-ray staining techniques, the variety of staining protocols for light microscopy extends far beyond what is currently possible in the field of X-ray imaging.

Striving for cellular resolutions with X-ray CT imaging, one fundamental bottleneck lies in artefacts due to mechanical instabilities and drifts. Cross-correlation based alignment techniques for CT reconstruction can correct for such artefacts but often require contrast-rich structures in the projection images^29^. Resin embedding weakens the sample-background contrast due to reduction of propagation phase effects and introduces higher noise levels. This raises the challenge of reconstruction alignment considerably and can render cross-correlation based methods unstable. By implementing our alternative alignment metric, we gain a significant amount of information (Fig. 6). This is particularly striking for the ring canal and the muscle fibre bundles in the high-resolution NanoCT slices (Fig. 6c,d). When applying this metric for correction of positional shifts, it has a similar effect on the results, as shown by cross-correlation based methods in the past^27,30^. Unlike these methods, however, our alignment routine is not restricted to merely compensating for positional shifts but can, in principle, correct for any desired geometry parameter^30^. Furthermore, it does not revolve around a parallel beam assumption^20,30^ and can thereby prove beneficial to a broader range of imaging systems.

These results reveal a multitude of attractive properties of the NanoCT for correlative microscopy. We envision the setup to find applications for VOI determination for FIB-SEM, as a reference for retrieving the original sample proportions in sectioning-based microscopy data, and particularly for multiscale approaches involving TEM.

To conclude, our work contributes to a deeper understanding of the 3D micromorphology of *L*. cf. *minuta* and holothurians in general and illustrates the advantages laboratory-based NanoCT can offer the field of correlative microscopy.

## Materials and Methods

### Specimen and sample preparation

The specimen of *Leptosynapta* cf. *minuta* was collected from samples of bottom sand near the Observatoire Océanologique de Banyuls-sur-Mer, France, in spring 2014.

The specimen was anesthetized with seawater isotonic magnesium chloride and fixed for several days in 2.5% glutaraldehyde in 1 mol/l cacodylate buffer, followed by post-fixation in 1% osmium tetroxide in 0.1 mol/l phosphate buffer. It was dehydrated in an ascending acetone series and embedded in Epon epoxy resin. To minimize the sample size for NanoCT acquisition, the resin block, which contained the specimen, was trimmed with a razor blade.

### X-ray NanoCT: setup and acquisition

The in-house-constructed table-top NanoCT setup does not use any X-ray or visible light optics and is based on the principle of a shadow microscope with geometric magnification^10^. The two main components are the nanofocus X-ray source (Nanotube prototype; Excillum)^46^ and a single-photon counting detector with a FOV of 1475 pixel × 195 pixel (PILATUS 300K-W 20 Hz, Dectris)^47,48^. The device can reach resolutions in the reconstructed tomography data down to 100 nm^10^.

The data sets were taken at a peak voltage of 60 kVp with 1,599 projections evenly distributed over an angular interval of 360°. In the overview NanoCT data the magnification was adjusted to achieve an effective voxel size of 540 nm with a FOV of 796 µm x 105 µm and an exposure time of 3 s per projection was chosen. This resulted in an acquisition time of ~2 h 45 min per CT scan. To extend the FOV in the vertical direction 12 CT data sets were acquired, which lead to a total duration of 33 h. In the high-resolution NanoCT data, an effective voxel size of 290 nm with a resulting FOV of 428 µm × 56 µm and an exposure time of 8 s was set. 7 CT data sets were acquired with a duration of ~5 h per CT scan which results in a total acquisition time of 35 h. The preparation of the CT acquisition, including the sample alignment in the setup, took approximately 3 h for both the overview and the high-resolution data set.

### X-ray NanoCT: image processing and reconstruction

For an enhanced soft-tissue contrast and to exploit the propagation-based phase-contrast effects, Paganin’s phase-retrieval algorithm was applied on the flat-field normalized projections, except for the data shown in Fig. S2a,c^22^. A mean energy of 20 keV, as detected by the X-ray camera for the set peak voltage, was used as input value and the attenuation and phase coefficient were chosen to optimize the image quality. The approach of Gureyev *et al*.^49^ was implemented for deblurring of the projections. The weighting factor alpha was adjusted to optimize the image quality.

The data sets were reconstructed with a state-of-the-art FBP routine. For the overview NanoCT data, only a centre shift correction was used. For the high-resolution NanoCT data, a sparsity metric to evaluate the alignment of the projections was employed^20^. Thereby, the detector offset was optimized iteratively using a gradient-free line-search algorithm to give the optimal response with respect to the metric. To reduce computational cost, this optimization was performed on a coarser grid and the results were transferred to the original resolution after using linear interpolation. For the comparative analyses of centre shift correction and the novel alignment routine, one high resolution NanoCT data set was additionally reconstructed with a conventional centre shift correction. After FBP reconstruction, the partial volumes along the longest axis of the specimen were combined. For this purpose, a 3D shift-vector was calculated by cross-correlation. For the overview NanoCT data set, 12 partial volumes were combined in 3D and for the high-resolution data 7 partial volumes were combined. These steps were implemented in python and carried out within an in-house-written python framework.

### SS-LM: sectioning

The specimen was sectioned for light microscopy at a thickness of 1 μm using a Histo Jumbo diamond knife (Diatome AG) in a MTXL ultramicrotome (RMC, Boeckeler Instruments) with ribbon formation of sections. The series was stained with methylene blue-azure II and afterwards sealed with resin and a cover slip (see^15^ for protocol of sectioning and staining).

### SS-LM: acquisition and image processing

The serially sectioned specimen of *Leptosynapta* cf. *minuta* was photographed with an Olympus DP73 microscope camera mounted on an Olympus BX53 compound microscope. For the overview image stack a 10x objective (0.4 NA) was used, resulting in a pixel size of 230 nm in the sectioning plane and a pixel length of 1 µm perpendicular to the sectioning plane. A 20x objective (0.75 NA) was used for the high-resolution image stack, which lead to a pixel size of 110 nm in the sectioning plane and again a pixel length of 1 µm perpendicular to the sectioning plane due to the slice thickness. With a dry lens and based on the assumption of a mean visible light wavelength of 450 nm, the Rayleigh criterion^50^ indicates a resolution in the sectioning plane of 686 nm in the overview data set and 366 nm in the high-resolution data set. The acquisition of the overview image stack, containing 1208 sections, took 2.5 h and of the high-resolution volume, containing 326 sections, took 1 h.

The image stack was converted into greyscales using Photoshop CS6 (Adobe) and adapted uniformly for better comparison with the NanoCT data. Very few missing slices where replaced by the previous or following section. For alignment, the image stack was imported into Amira (Thermo Fisher Scientific). Thereby, the ‘AlignSlices’-module was used with manual corrections of individual slices.

### Co-registration of NanoCT and SS-LM data, segmentation and visualization

The co-registration, segmentation and visualization was performed with Amira 6.4 (Thermo Fisher Scientific). The grey values of the ss-LM data were inverted in Amira to match the grey values of the NanoCT data. For co-registration of the overview and the high-resolution data from NanoCT and ss-LM, the LM volume was stretched perpendicular to the sectioning plane to account for the non-isotropic nature of the ss-LM data and aligned manually to roughly match the NanoCT data as a first approximation. For optimized alignment, the ‘Register Images’-module in Amira 6.4 was used. Thereby, the option of ‘Transform: Aniso-Scale’ was chosen to enable the ‘Register Images’-tool to converge toward a valid result. Nevertheless, due to the unevenly distributed distortion artefacts within the ss-LM volume, there are still some mismatches in the co-registration. After the co-registration, the ss-LM data was resampled. Movie S1 was acquired under a light microscope and edited with Shotcut (Meltytech, LLC) to correct for vibrations. Movie S2 and S3 were generated with Amira 6.4.

### CNR analysis and line-profile plots

The data analysis was performed with self-written python code. For the results, presented in Figs. 4 and 5, a LM image in the sectioning plane and a NanoCT slice, as shown in Fig. 3f,e, were selected.

For the CNR calculation (Fig. 4), 5 ROIs were defined in both images. Thereby, regions were chosen with respect to maximum homogeneity, containing as little sub-structures as possible, and thereby avoiding falsely increased noise levels. In both the NanoCT slice and the LM image, the ROIs were placed in corresponding regions with the same tissue type. As a reference, the mean value of the resin background ROI was set to 0. The CNR was calculated according to the following equation

${\rm{CNR}}=({{\rm{\mu }}}_{{\rm{ROI}}}-{{\rm{\mu }}}_{{\rm{ref}}})/\sqrt{{{\rm{\sigma }}}_{{\rm{ROI}}}^{2}+{{\rm{\sigma }}}_{{\rm{ref}}\,}^{2}}$, whereby ${{\rm{\mu }}}_{{\rm{ROI}}}$ denotes the mean value in the respective ROI and ${{\rm{\mu }}}_{{\rm{ref}}}$ the mean value in the reference material, here the resin. ${{\rm{\sigma }}}_{ROI/ref}$ represents the standard deviation from the mean value in the respective ROI or the reference material.

For the line profiles of Fig. 5, two perpendicular lines were chosen in both images. Thereby, attention was paid to include the same structural details as far as possible. However, this was not perfectly feasible due to slight deviations in the co-registration. For better comparability, the obtained grey value distributions were normalised with the maximum grey value in each line plot and the resin background was subtracted.

## Supplementary information

Supplementary Information.

Supplementary Information2.

Supplementary Information3.

Supplementary Information4.

## Data Availability

The data that support the findings of this study are available on request from the corresponding author S.F.
