# Supplementary material for: Nanoscopic X-ray tomography for correlative microscopy of a small meiofaunal sea-cucumber
Source: Sci Rep. 2020 Mar 3;10:3960. doi: 10.1038/s41598-020-60977-5 (PMC7054411; doi:10.1038/s41598-020-60977-5)
Supplement: Supplementary file 4 — Supplementary Information4. [file 41598_2020_60977_MOESM4_ESM.pdf]

Supplementary Information

Nanoscopic X-ray tomography for correlative microscopy of a small meiofaunal sea-cucumber

Simone Ferstl[1*, †], Thomas Schwaha[2, †], Bernhard Ruthensteiner[3], Lorenz Hehn[1], Sebastian Allner[1], Mark Müller[1], Martin Dierolf[1], Klaus Achterhold[1] and Franz Pfeiffer[1, 4]

1. *Chair of Biomedical Physics, Department of Physics and Munich School of BioEngineering, Technical University of Munich, Boltzmannstraße 11, 85748 Garching, Germany.*
2. *Department of Integrative Zoology, University of Vienna, Althanstraße 14, 1090 Vienna, Austria.*
3. *Zoologische Staatssammlung München - SNSB, Münchhausenstraße 21, 81247 Munich, Germany.*
4. *Department of Diagnostic and Interventional Radiology, School of Medicine and Klinikum rechts der Isar, Technical University of Munich, Ismaninger Straße 22, 81675 Munich, Germany.*

†.: These authors contributed equally to this work.

*** Corresponding author:**
Simone Ferstl, Chair of Biomedical Physics, Department of Physics and Munich School of BioEngineering, Technical University of Munich, Boltzmannstraße 11, 85748 Garching, Germany, phone: +49 (0)89 289 10820, email: simone.ferstl@tum.de.

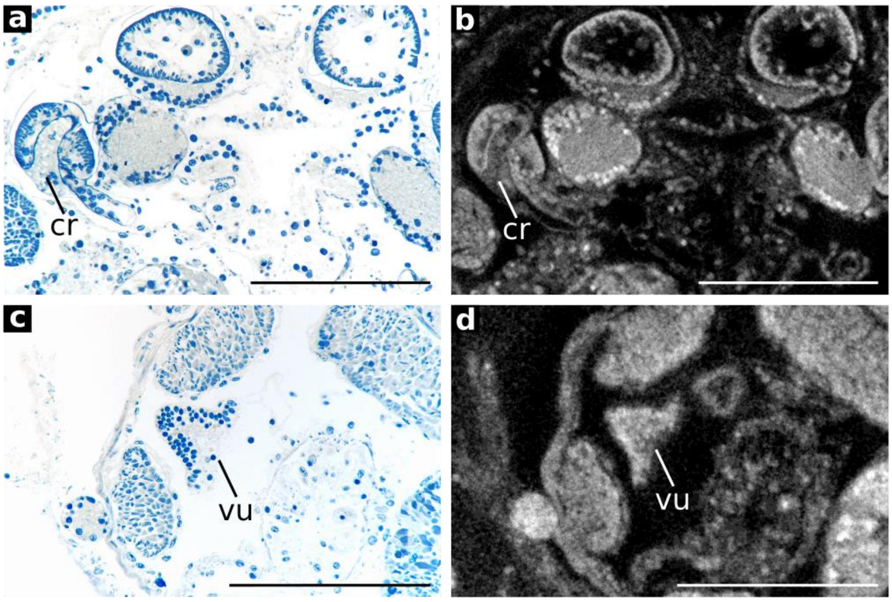

Fig. S1. Comparison between ss-LM images and NanoCT data. **(A)** LM section with the calcareous ring (cr). **(B)** Corresponding high-resolution NanoCT slice (effective voxel size ~290 nm) with the calcareous ring (cr). **(C)** LM section with the vibratile urn (vu). **(D)** Corresponding overview NanoCT slice (effective voxel size ~540 nm) with the vibratile urn (vu). Legend: cr: calcareous ring, vu: vibratile urn. Scalebars: 100 µm.

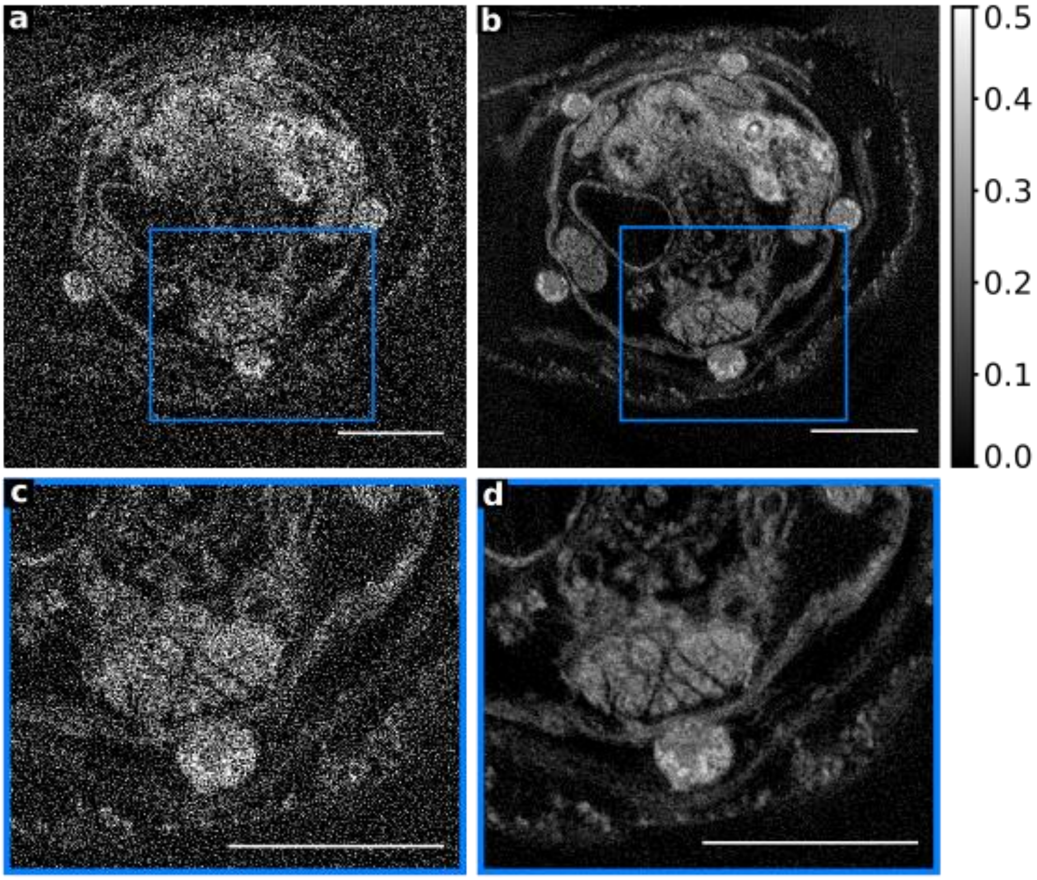

Fig. S2. Comparison of filtered-back-projection (FBP) reconstructed high-resolution NanoCT data (effective voxel size ~290 nm) without and with propagation-based phase retrieval. **(a)** FBP reconstructed slice of the high-resolution NanoCT data. **(b)** FBP reconstructed slice with propagation-based phase retrieval applied to the projections of the high-resolution NanoCT data. **(c)** Detail image of the blue marked region of interest (ROI) in **(a)**. **(d)** Detail image of the blue marked ROI in **(b)**. Scalebars: 100 µm.

Movie S1: Recording of the alive specimen taken under a light microscope.

Movie S2: Animation illustrating the overview ss-LM and NanoCT data and the segmented organs, which are shown in **Fig. 1** and **Fig. 2**.

Movie S3: Animation illustrating the high-resolution ss-LM and NanoCT data, which are shown in **Fig. 3** and **Fig. 4**, and showing the position of the data sets within the overview data.
